# Complex sublinear burrows in the deep sea may be constructed by amphipods

**DOI:** 10.1002/ece3.9867

**Published:** 2023-03-16

**Authors:** Angelika Brandt, Chong Chen, Anne Helene S. Tandberg, Olmo Miguez‐Salas, Julia D. Sigwart

**Affiliations:** ^1^ Department of Marine Zoology Senckenberg Research Institute and Natural History Museum Frankfurt Germany; ^2^ Faculty of Biological Sciences, Institute of Ecology, Diversity and Evolution Goethe University Frankfurt Germany; ^3^ X‐STAR Japan Agency for Marine‐Earth Science and Technology (JAMSTEC) Yokosuka Japan; ^4^ Department of Natural History University Museum, University of Bergen Bergen Norway

**Keywords:** Amphipoda, Bering Sea, Hadzioidea, life trace, trypophobia

## Abstract

Trails, burrows, and other “life traces” in sediment provide important evidence for understanding ecology—both of the maker and of other users—and behavioral information often lacking in inaccessible ecosystems, such as the deep sea or those that are already extinct. Here, we report novel sublinear rows of openings in the abyssal plains of the North Pacific, and the first plausible hypothesis for a maker of these constructions. Enigmatic serial burrows have now been recorded in the Pacific and Atlantic deep sea. Based on image and specimen evidence, we propose that these Bering Sea excavations represent amphipod burrows, while the maker of the previously known Mid‐Atlantic Ridge constructions remains undetermined. We propose that maerid amphipods could create the Pacific burrows by eating–digging horizontally below the surface along a nutrient‐rich layer in the sediment, making the serial openings above them as they go, for conveniently removing excavated sediment as the excavation progresses. These striking structures contribute to local biodiversity, and their maker could be considered a deep‐sea ecosystem engineer.

## Graphical abstract

Enigmatic linear series of holes in the deep sea have now been recorded in the Pacific and Atlantic. We propose that these Bering Sea burrows are constructed by amphipods.

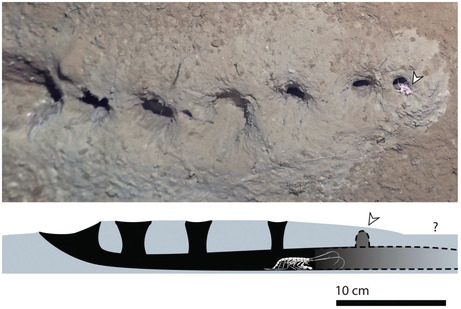

## 1 INTRODUCTION

Animals make burrows and nests for shelter, hibernation, reproduction, food storage, and other reasons. Some organisms build complex and (relatively) colossal structures, such as termite nests and beaver dams, which reveal their engineering capabilities and intricate social lives. These are not only important for the makers: secondary and even tertiary uses of such structures are common (Noonan et al., 2015). How complex life traces are built and used is significant for understanding both the maker and broader ecosystem function.

In the abyssal plains, the largest habitat surface on Earth, limited observation opportunities and the infaunal lifestyle of most animals combine to make direct observation of ecology and behavior very challenging. As such, life traces are often the only opportunity to understand the ways of life on the deep seafloor. Observations of seafloor traces and burrows are abundant (Ewing & Davis, 1967), yet it is usually unclear which animals produce or are associated with what traces.

**FIGURE 1:**
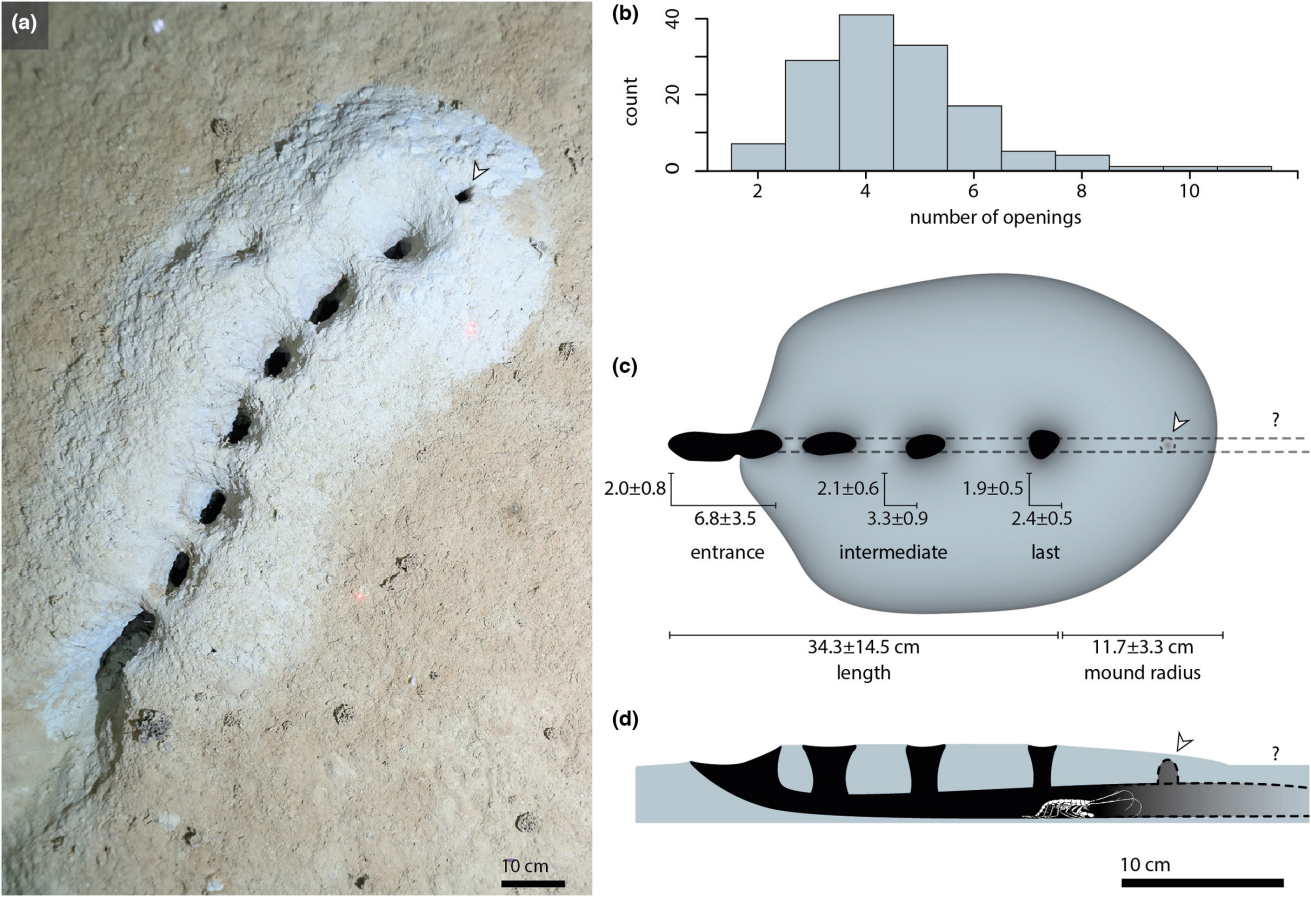
Characteristics of the Bering Sea burrows. (a) Example burrow with eight openings, entrance positioned at bottom; laser points are 40 cm, scale bar 10 cm. (b) Frequency of observation of burrows of different numbers of openings. (c) Drawing of a typical burrow indicating the mean and standard deviation of main dimensions for entrance opening, intermediate openings, and last opening, total length of the series from entrance to last opening, and terminal radius of the mound of excavated sediment (all measurements in cm). Dashed lines indicate inferred horizontal shaft and potential space continued beyond the last opening. (d) Schematic interpretation of cross‐section for the burrow seen in (c). Scale bar refers to (c) and (d).

One of the most enigmatic seafloor traces is the sublinear series of openings (sometimes referred to as “pogo stick trails”; Bell et al., 2013) known since the 1960s but only from the Mid‐Atlantic Ridge (MAR) near the Azores (Ewing & Davis, 1967; Heezen & Hollister, 1971; Hinga, 1981; Vecchione & Bergstad, 2022). We serendipitously discovered superficially similar structures during a recent expedition in the Bering Sea, at depths around 3500 m. At first glance, these lines of openings within a sublinear mound resemble the dorsal configuration of chitons (Figure 1a). Here, we examine our new finding in comparison to the enigmatic traces in the Atlantic, and historical observations of potential burrow makers. We can use these comparisons to consider whether these burrows represent a global phenomenon, and what roles they might play in an abyssal ecosystem.

## 2 METHODS

The “AleutBio” expedition (July–September 2022) on board R/V *SONNE* was designed to systematically investigate deep‐sea fauna across all size classes in the Bering Sea and Aleutian Trench, with onboard expertise for most major animal groups. We conducted three towed camera transects, each approximately 0.5 nmi (~0.9 km), with a visible width of approximately 1.85 m, between 53–55°N and 172–175°W, at depths between 3507–3653 m (Table S1). The Ocean Floor Observation System on board R/V *SONNE* is equipped with a Full‐HD video camera and a mirrorless camera with a resolution of 8192 x 5464 pixels (Canon EOS R5), plus fixed laser points to scale still images.

We use the term “burrow” here to refer to the target structures, a distinct set of oval crater rows, comprising a sublinear series of two or more openings surrounded by a mound of excavated sediment. These excavation structures were counted from video, and stills were used for measurements and to identify fauna inside the burrows. Because these were all captured by a towed camera system, each burrow was seen only for a matter of seconds. Within a burrow, the sequence and directionality of construction could be clearly inferred morphologically (see below). We refer to the oldest opening as the “entrance” opening, the most recent as “last,” and others as intermediate openings. The following measurements were taken in ImageJ v.15.3 k for burrows that were completely visible and in focus in still images: length and width of each opening, burrow length (distance from the entrance to the last opening), and mound radius (radius of excavated sediment around the last opening). The mound radius was measured from the final opening to the edge of the discolored, excavated sediment. Openings toward the entrance would have been constructed earlier, so the terminal mound radius was chosen to limit variability from potential erosion or slumping that appeared common toward the entrance. We also attempted to identify and count animals seen on the burrows, either within the openings or on the surrounding mound.

## 3 RESULTS

Each burrow represents a connected excavated structure with multiple openings apparently connected below the surface; the openings are surrounded by a mound of excavated sediment. The openings are subcircular, with the longer axis oriented with the axis of the overall series, and the series is usually slightly curving. The surrounding mound is asymmetrically distributed, formed of fusing piles slumping around each opening, but overall oriented away from the “entrance” opening toward the last opening and terminating in a round pile of fresh sediment around the last opening.

In total, 196 burrows were seen in the Bering Sea in a seafloor area of approximately 5660 m^2^, and none in the Aleutian Trench. Still images captured 146 burrows of which 74 could be used for detailed measurements. Their average length was 34.3 cm (Figure 1; Table S2). Number of openings could be counted on 139 burrows, ranging between 2–11 and averaging 4.5 (Figure 1b **).** A series of mud bridges separated the openings, occasionally evidence of collapse was seen where only two lateral projections remain. Fresh‐looking excavated sediment, more pale or gray than the surface, forms a pile around each opening, each fusing with the previous to create the continuous surrounding mound of the burrow series. Still images occasionally provided clear visuals on the internal structures of the excavations, showing a complete lack of backfilling within. The burrows have a clear directional orientation indicated by the shape of the surrounding mound. Around the entrance, the mound was mostly covered by surface sediment and not clearly elevated, and around the “last” opening, the mound has a clear elevation and edge with a circular contour (average radius 11.7 cm) which indicates this as the site of most recent active excavation.

The entrance end was usually associated with a more elongated opening, sometimes formed by two openings fusing, and a sloped trough on the distal end indicative of frequent traffic, which is the basis for the “entrance” term in our description. The entrance opening was typically the longest, and the intermediate openings were compressed ovals and larger than the last opening which was also more rounded. The width of all openings remained similar within the series comprising a given burrow and across separate burrow structures. All openings in a burrow were connected in a single subsurface horizontal shaft. In some burrows, we had visuals that the subsurface passages between the openings were much taller than wide and also narrower than the adjacent surface openings. Some views on video indicated the presence of a further extension of the horizontal shaft beyond the last opening, continuing an unseen part of the burrow.

**FIGURE 2:**
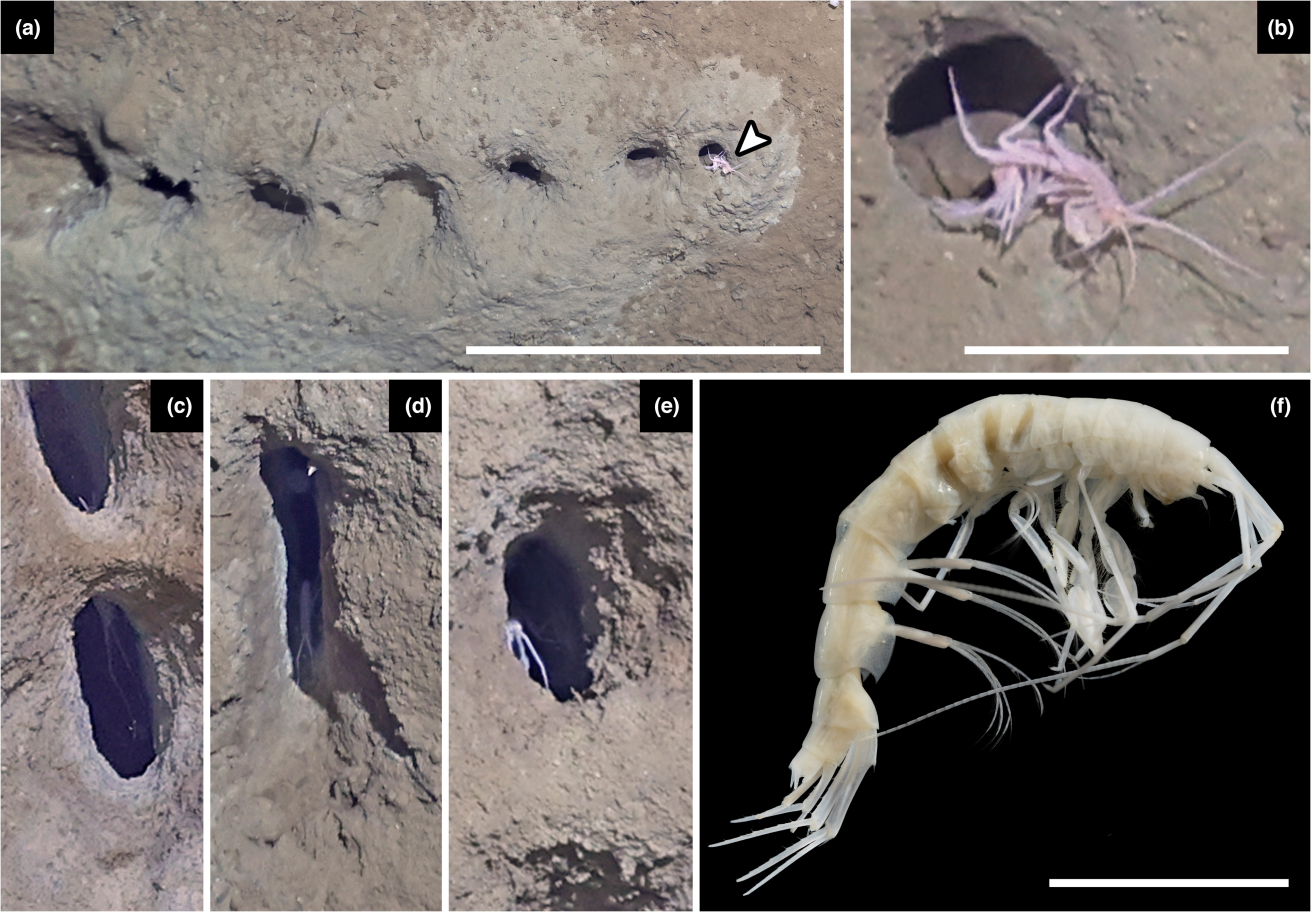
The proposed burrow maker. (a) Complete burrow showing a maerid amphipod at the last opening, potentially in process of excavation (53°47.959′N, 173°38.152′W, depth 3590 m, 30 July 2022). (b) Enlarged view of maerid amphipod seen in A. (c‐e) Observations of other likely maerid amphipod individuals within burrows. (f). Photograph of the collected specimen (54°32.14′N; 172°33.54′W, depth 3500 m, 27 July 2022). Scale bars, (a) 40 cm; (b) 5 cm; (f) 1 cm.

**FIGURE 3:**
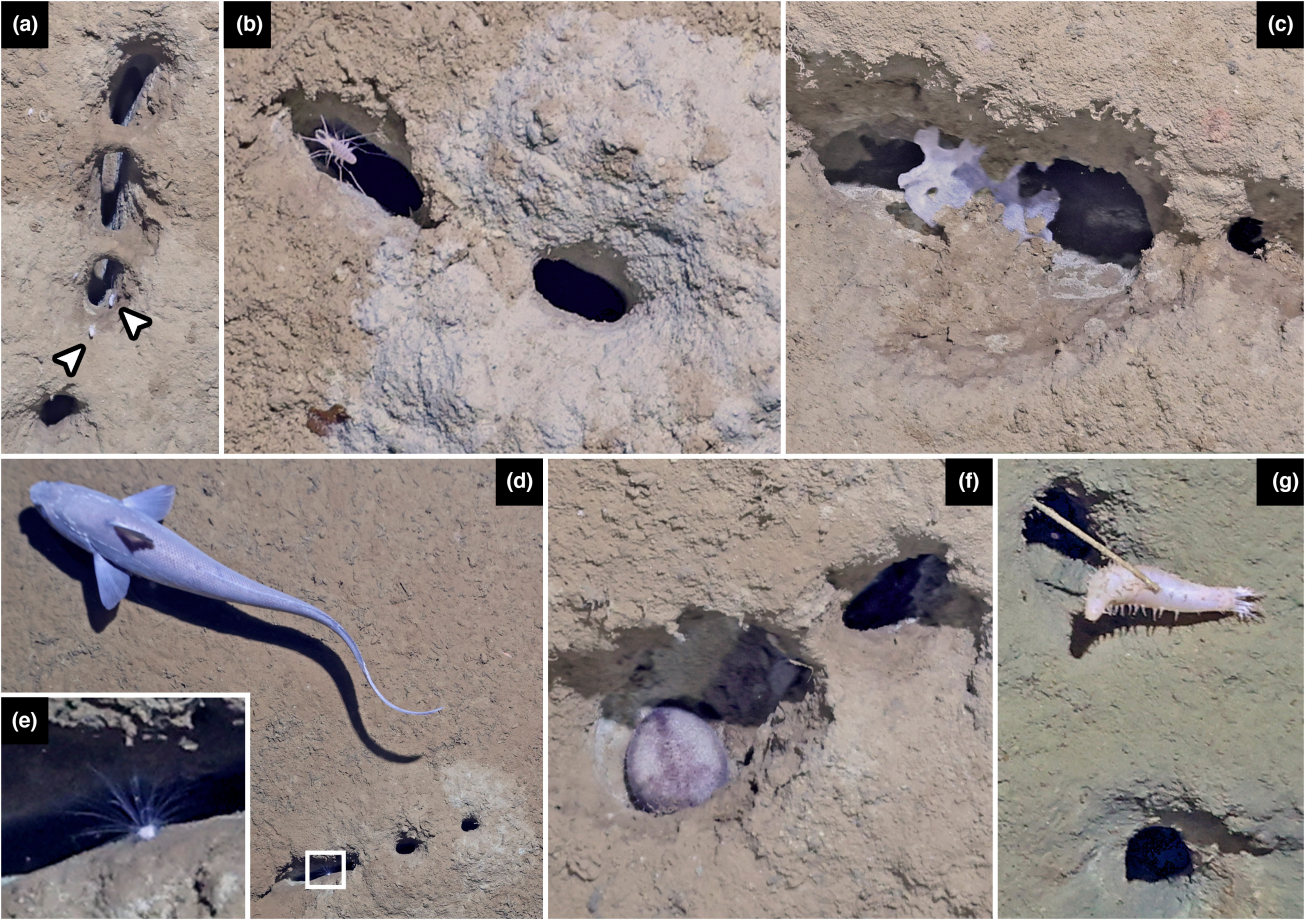
Fauna associated with burrow openings. (a) Munnopsid isopods (arrowheads), (b) Desmosmatid isopod, (c) Glass sponge, (d) Giant Grenadier, the main potential local predator, with inset (e) probable long‐spined aspidodiadematid urchin, (f) Irregular urchin (possibly *Cystechinus* sp.), and (g) Holothurian, *Abyssocucumis*.

A total of 96 animals were found in proximity to the burrows in still images (Figures 2 and 3; Table S3), including invertebrates on and within the burrow openings, and Giant Grenadier fishes (*Albatrossia pectoralis*) were frequently seen roaming around the burrows.

## 4 DISCUSSION

Enigmatic, long sublinear series of openings known from the MAR (Vecchione & Bergstad, 2022) represent the only obvious point of comparison to these new Bering Sea burrows; however, there are some distinct differences. The MAR “pogo stick trails” have been recorded in several locations between 1471–2082 m depth (Ewing & Davis, 1967; Heezen & Hollister, 1971; Hinga, 1981; Vecchione & Bergstad, 2022), which are much shallower than our stations at around 3500 m. The openings are of similar size, or slightly smaller in the MAR (diameter ~1.5 cm *fide* Vecchione & Bergstad, 2022). The MAR openings are round or slit like, but consistently shaped within a series, described as a series of crater cones (Heezen & Hollister, 1971); by contrast, the Bering Sea burrows contain shorter sequences of openings, always with a clear directional orientation, and a mound that surrounds and delimits the burrow as a unit. Recent observations of the MAR structures also could not confirm any horizontal connectivity between the openings in the series and noted a lack of associated fauna in the openings (Vecchione & Bergstad, 2022). The surface structure of the Bering Sea burrows is sufficiently distinct to qualify as a novel structure, not a new occurrence of the MAR traces, and likely with a different maker. This potential convergence of structures in different ocean basins, such as in simpler worm, crustacean, and bivalve burrows, is an important caution for the interpretations of trace fossils.

We observed a diverse fauna associated with the novel burrows, which provided an initial set of candidate makers. Fishes make complex and sometimes geometrically arranged nests but usually not linear (Seike & Yamashita, 2022), and the Grenadiers frequently observed are too large to enter the narrow burrow entrance. Likewise, we observed urchins in the burrow entrance, but it is clear that they could not enter the narrow passage into the horizontal shaft and no backfilling was observed. Isopods were frequently observed within burrow openings (Table S3), but they generally lack suitable appendages for digging (Table S4). Annelid worms frequently make burrows and tubes, but a vermiform body shape does not correspond to the apparently trampled trackways of excavated sediment, especially the tall canal‐like structure frequently seen extending the entrance opening. Finally, we also observed one type of animal from a group known to make burrows, with a body size corresponding to the opening diameter, a narrow body, and enlarged appendages used for digging: amphipods.

In one image, we clearly see an amphipod most likely in the family Maeridae, probably a species of *Maera* or a closely related genus, probably in the act of carrying sediment from the burrow (Figure 2a); similar large amphipods were seen within the burrows several times (Figure 2b‐d). This prompted a moment of *déjà vu* for one of us (AB), recollecting another unexpected observation of burrowing in Maeridae. In the 1980s, Oliver Coleman investigated burrowing and grooming behavior of *Paraceradocus gibber* from Antarctica in a cooling container, and revealed that the species' large gnathopods were not used for predatory behavior, as was thought at the time, but rather for shoveling sediment to construct holes under stones (Coleman, 1989). In many amphipods, large gnathopods have additional functions such as fighting or mating and can be sexually dimorphic (Conlan, 1991). Burrowing in *P. gibber* was accompanied by a backward somersault due to the long first and second antennae (Video S1). Coleman (1989) reported one other maerid species from the North Atlantic, *Maera loveni*, burrowing in the same manner, but in soft sediment where it feeds on detritus (Atkinson et al., 1982; Coleman, 1989; Enequist, 1949).

Although several *Maera* species are known from the Bering Sea region, they are all coastal or shallow water (Krapp‐Schickel & Jarrett, 2000), except the seemingly wide‐spread *Maera loveni* recorded from the Bering Sea down to 300 m, and at 1200 m in the Norwegian Sea (Krapp‐Schickel & Vader, 2013). We collected a specimen of *Maera* sp. via epibenthic sledge from a station where we observed the burrows (Figure 2e), clearly distinct from *M. loveni* and likely undescribed. The collected specimen (now in the Senckenberg Museum, Frankfurt) is smaller than the one observed exiting the burrow (Figure 2b), but they are morphologically similar and likely belong to the same species.

Several amphipods are known to make burrows, the majority of which have long and narrow bodies with relatively large gnathopods, long antennae, and strong uropods. This is likely a convergent functional morphology that has evolved multiple times across amphipods in several families (Table S4). Buhl‐Mortensen and colleagues (Buhl‐Mortensen et al., 2016) observed the deep‐water unciolid *Neohela monstrosa* also using gnathopods and antennae to shovel out sediments from burrows in the Norwegian and Barents seas. These burrows seem to be much smaller and less structured in placement, but they support the same basic use. Apart from *N. monstrosa*, all documented burrow‐making amphipods are from relatively shallow waters, but this most likely represents a significant sampling bias.

Some burrowing amphipods dig down to a nutritious sediment layer, and then eat–dig a horizontal tunnel (Enequist, 1949). Burrow depth may correspond with body size, deeper in larger amphipods (Thiel, 1999). The size and shape of the entrance and the slit‐like connections of Bering Sea burrows match the maerid amphipods observed and sampled. Amphipod burrows might be used for extended parental care (Thiel, 1999)—a potential function for the burrows observed herein since the maerid *Paraceradocus gibber* exhibits parental care and multigenerational co‐habitation (Coleman, 1989).

The overall morphology of these excavations may provide other benefits for flow or sediment trapping, similar to the passive ventilation generated by hexagonal seafloor traces whose maker remains unclear (e.g., Rona et al., 2009). Considering the amount of sediment excavated, the practical function of the sublinear openings is likely to be points where the amphipod pushes out sediments from extending the excavation. The openings are all of a very similar width; the last opening is usually smaller because it is a work in progress. The entrance opening clearly has compacted sediment that indicates longer usage, for animal traffic if not for sediment removal, compared to the increasingly freshly excavated sediment surrounding each opening further down the line of openings. By making new openings as they continue to dig, the makers would benefit by not having to transport the sediment to the entrance opening.

Functional comparisons with other amphipods suggest these deep‐sea amphipods would be capable of creating a burrow. The size, regular geometry, and known complexity of these structures are considerably beyond what is documented for amphipod constructions to date.

These burrows potentially reveal unexpected complexity of behavior in amphipods. It also highlights their ecological importance to the Bering Sea abyssal plains, where the burrows are apparently used by other fauna for potential functions such as predator avoidance (Figure 2). We note that there were more burrows at the two sampling stations with higher megafaunal species richness and relatively few burrows in the station with lower biodiversity (Table S3; Sigwart et al., 2023). Earlier work emphasized the importance of small‐scale heterogeneity as a driver of deep‐sea biodiversity (Jumars, 1976; Levin et al., 1997) large excavations such as these could persist for long time spans in the deep sea, and provide distinct and persistent niches from microbes to macrofauna.

Bioturbation is important to benthic biodiversity at multiple scales, starting from the Cambrian Substrate Revolution, and the burrows reported here may contribute to supporting biodiversity in the Bering Sea. Elevated mounds of compacted excavated sediment provide three‐dimensional complexity to the abyssal plain, and makers of these burrows are deep‐sea ecosystem engineers. Traces remain important evidence for understanding ecology, both of the maker and other users—a layer of information much lacking in inaccessible ecosystems, such as the deep sea or those that are already extinct.

## AUTHOR CONTRIBUTIONS

**Angelika Brandt:** Conceptualization (equal); funding acquisition (equal); investigation (equal); resources (equal); writing – original draft (equal); writing – review and editing (equal). **Chong Chen:** Conceptualization (equal); investigation (equal); methodology (equal); visualization (equal); writing – original draft (equal); writing – review and editing (equal). **Anne Helene S Tandberg:** Investigation (equal); visualization (equal); writing – original draft (equal); writing – review and editing (equal). **Olmo Miguez Salas:** Investigation (equal); methodology (equal); visualization (equal); writing – review and editing (equal). **Julia Sigwart:** Conceptualization (equal); data curation (equal); formal analysis (equal); investigation (equal); methodology (equal); visualization (equal); writing – original draft (equal); writing – review and editing (equal).

### OPEN RESEARCH BADGES

This article has earned Open Data, Open Materials and Preregistered Research Design badges. Data, materials and the preregistered design and analysis plan are available at [10.5061/dryad.9s4mw6mm7].

## Supporting information


Table S1

Table S2

Table S3

Table S4

Video S1

## Data Availability

All still images captured from the three sampling stations are available via Dryad: https://datadryad.org/stash/share/X2cdNYK65Caisfb6cyVhLecFI1aoaCYaLbC4NJfoQsk
