# Supplementary material for: Complex sublinear burrows in the deep sea may be constructed by amphipods
Source: Ecol Evol. 2023 Mar 16;13(3):e9867. doi: 10.1002/ece3.9867 (PMC10018091; doi:10.1002/ece3.9867)
Supplement: Supplementary file 1 — Table S1 [file ECE3-13-e9867-s005.pdf]

Supplementary Table S1. Location and depth of stations where burrows were observed: USBL (seafloor) position data for the Ocean-Floor Observation System (OFOS) at three stations where burrows were observed and ship position data for epi-benthic sledge (EBS) deployment that collected the maerid amphipod specimen. Transects of the OFOS were approximately 1 km each, transect of the EBS was approximately 2 km.

	Time (UTC)	Latitude	Longitude	Depth (m)
**OFOS (equipment position)**				
**Station 1 Start**	28/07/2022 02:05	54− 33.219' N	172− 34.922' W	3512
**Station 1 End**	28/07/2022 04:10	54− 33.536' N	172− 34.273' W	3507
**Station 2 Start**	29/07/2022 02:58	54− 32.158' N	174− 37.532' W	3650
**Station 2 End**	29/07/2022 04:43	54− 32.498' N	174− 36.825' W	3653
**Station 3 Start**	30/07/2022 23:30	53− 48.314' N	173− 38.258' W	3588
**Station 3 End**	31/07/2022 01:32	53− 47.690' N	173− 38.262' W	3587
**EBS (ship position)**				
**Station 1 Start (deployment)**	27/07/2022 16:51	54− 31.423' N	172− 36.583' W	3516
**Station 1 (EBS at seafloor)**	27/07/2022 18:46	54− 32.142' N	172− 33.544' W	3499
**Station 1 (end of transect)**	27/07/2022 19:48	54− 32.527' N	172− 31.923' W	3502
**Station 1 End (equipment on deck)**	27/07/2022 22:04	54− 33.536' N	172− 34.273' W	3504
