# Supplementary material for: Complex sublinear burrows in the deep sea may be constructed by amphipods
Source: Ecol Evol. 2023 Mar 16;13(3):e9867. doi: 10.1002/ece3.9867 (PMC10018091; doi:10.1002/ece3.9867)
Supplement: Supplementary file 2 — Table S2 [file ECE3-13-e9867-s003.pdf]

Supplementary Table S2. Details of the 74 burrows examined for dimensional measurements.

		Entrance opening (HV)		Intermediate openings (IV)														Tail opening (TV)				
**Burrow ID**	**Openings**	**HV Length**	**HV Width**	**IV1 Length**	**IV1 Width**	**IV2 Length**	**IV2 Width**	**IV3 Length**	**IV3 Width**	**IV4 Length**	**IV4 Width**	**IV5 Length**	**IV5 Width**	**IV6 Length**	**IV6 Width**	**IV7 Length**	**IV7 Width**	**TV Length**	**TV Width**	**End Radius**	**End Width**	**Burrow length**
1_68	6	3.07	2.88	3.94	3.33	3.04	1.84	2.32	1.95	2.45	2.4							1.41	1.52	11.04	27.45	49.52
1_73 right	7	n	n	3.57	1.55	3.09	1.6	3.57	2.24	3.04	1.79	3.04	2.27					2.03	2.11	14.39	25.67	n
1_92	3	3.2	1.81	2.4	2													2.51	2.16	10.64	23.43	23.3
1_94	4	n	n	2	2.11	2.37	2.37											2.51	2.32	13.09	22.5	n
1_106 left	4	7.25	1.6	3.49	1.44	2.51	1.33											2	1.52	10.98	21.11	25.86
1_140	4	9.76	2.99	2.85	1.76	2.21	1.76											2.08	2	11.67	25.22	34.38
1_252	4	7.76	1.92	5.81	2.21	2.75	2.61											2.24	2	13.17	25.62	37.98
1_260	4	n	n	3.41	1.76	2.83	1.44											2.72	1.68	11.99	25.08	n
1_270	6	5.46	3.01	4.02	2.29	3.71	1.87	2.8	1.79	2.56	1.68							1.95	1.55	10.1	21.75	48.14
1_305	6	3.65	1.87	3.57	1.39	2.91	1.39	2.8	1.79	3.23	1.55							2.77	1.97	10.8	26.97	34.86
1_307	5	5.44	2.85	3.44	2.51	2.64	2.4											2.77	1.87	13.38	25.27	n
1_309	5	4.53	2.61	4.21	2.4	3.07	1.65	2.8	1.65									2.05	1.63	13.94	27.03	37.98
1_311	7	n	n	3.36	1.71	2.43	1.71	3.47	1.89	3.04	1.76	2.53	2.27					2.13	2.03	11.51	23.7	n
1_361 right	5	n	n	2.4	1.87	2.27	1.97	2.37	2.08									2.69	1.52	12.85	26.07	n
1_414	5	6.08	1.71	3.31	1.44	2.13	1.44	1.79	1.81									1.25	1.97	15.06	28.31	39.1
1_444	8	n	n	5.44	2.77	5.81	3.07	5.14	2.88	4.34	2.91	5.04	2.43	3.84	2.83			2.53	1.89	11.91	41.37	n
1_482	3	3.07	1.81	2.96	1.57													2.19	1.39	7.89	15.06	21.14
1_507	2	4.34	1.71															2.96	1.47	9.06	17.33	11.01
1_554	8	3.55	2.19	2.29	1.55	3.33	2.11	3.01	1.95	2.4	1.65	2.56	2.08	2.4	1.89			2.27	2.08	16.42	32.39	60.88
1_580	4	12.45	2.19	4.48	2.19	3.57	2.24											2.35	1.81	14.9	27.37	42.99
1_599	4	4	2.61	3.52	2.29	3.89	2.45											3.09	2.59	15.41	28.25	33.21
1_609	4	11.67	1.68	3.44	2.56	3.36	2.48											2.4	2.27	15.3	33.64	44.51
1_618	4	n	n	4.53	3.81	3.33	2.85	3.71	2.16									2.56	1.25	14.69	32.52	43.74
1_622	5	16.9	1.76	3.55	2.32	3.23	1.92	3.52	2.13									2.48	2.19	18.34	32.09	51.36
1_629	3	5.65	1.97	3.6	1.52	2												1.44	0.95	18.5	21.24	20.66
1_640	n	n	n	5.57	3.65	4.98	2.64	3.71	2.99									3.86	2.37	17.35	38.09	n
1_664	2	5.25	1.39															2.11	1.36	11.51	20.04	12.69
1_685	4	n	n	3.76	3.49	4.66	4.21	3.49	2.75									2.4	2.13	11.22	21.88	n
1_729 left	6	n	n	3.44	2.83	3.23	2.35	3.52	2.13	3.15	2.35							3.68	2.37	11.43	28.28	n
1_731	5	n	n	4.16	3.12	3.36	2.72	3.92	3.12									2.56	2.48	15.59	29.83	n
1_762	4	3.78	1.23	2.37	1.17	2.64	1.39											2.03	1.52	9.54	18.23	25.11
1_774	3	6.37	1.39	3.36	1.36													2.64	1.71	11.78	23.46	20.74
2_258	3	6.48	1.36	2.24	1.17													2.24	1.39	8.53	17.67	16.69
2_265	4	n	n	2.77	2.24	2.51	2.03	2.64	1.87									1.65	1.28	10.13	21.75	36.33
3_45	5	7.65	1.95	4.93	1.89	3.73	1.57	3.07	1.97									2.45	2.16	9.44	21.54	39.93
3_53	8	n	n	5.33	2.91	4.96	3.44	4.45	3.28	3.55	2.64	3.41	2.32	3.55	2.83	2.53	2.93	2.32	2.32	11.46	24.71	72.07
3_70	3	8.05	2.21	3.86	2.53													2.91	2.32	11.14	22.84	23.32
3_79	5	2.43	1.47	3.89	1.47	2.67	1.71	2.61	1.95									1.89	1.73	9.3	22.55	30.09
3_98	4	n	n	3.84	2.29	3.28	2.24											2.64	2.4	9.6	21.03	n
3_100	3	6.32	1.36	2.37	2.08													1.65	1.6	8.37	17.46	20.55
3_202	6	2.48	0.91	4.61	0.99	3.17	1.07	2.11	1.33	2.16	1.63							1.95	1.33	7.54	16.69	40.44
3_211	3	7.73	1.63	2.37	1.89													1.87	1.6	6.32	15.62	23.19
3_233	4	6.93	1.25	2.35	1.36	2.43	1.55											1.97	1.49	9.86	22.95	28.17
3_250	5	n	n	2.96	2.45	2.61	1.95	2.32	1.84									2.75	1.41	12.13	26.25	n
3_307 right	5	8.02	1.36	3.57	1.84	2.93	1.76	2.67	1.47									2.67	1.76	12.93	25.22	36.78
3_313	5	8.34	1.31	3.89	1.17	3.12	1.92	2.96	1.92									2.99	2.48	12.66	27.67	37.16
3_317 botto	7	9.62	3.28	3.12	1.76	3.04	1.92	3.76	2.24	3.71	2	2.56	2.56					2.32	2.24	7.81	21.16	57.36
3_330	3	7.2	1.68	4.48	1.84													3.52	1.84	13.3	21.91	22.23
3_352 botto	6	19.64	5.62	2.08	2.08	5.62	2.56	4.72	2.16	3.71	2.64							2.8	2.64	22.18	43.21	76.82

Supplementary Table S2. Details of the 74 burrows examined for dimensional measurements.

3_384	3	3.86	1.84	2.77	1.57							2.11	1.47	5.92	13.59	18.66
3_394	5	n	n	4.74	3.41	3.63	2.56	4.58	2.99			3.6	2.32	13.91	32.87	n
3_415	3	11.49	1.15	3.07	1.31							2.29	1.6	9.78	21.96	24.44
3_421	4	11.04	2.35	3.92	2.11	3.31	2.4					2.32	1.81	9.94	26.2	38.52
3_461	4	6	2.67	4.13	2.64	3.68	2.56					2.64	2.51	14.47	31.05	32.68
3_470	3	n	n	2	1.33							2.43	1.07	5.46	12.53	n
3_492 boton	4	5.28	1.41	2.56	1.55	2.99	1.39					2.19	1.23	7.73	15.03	27.51
3_493 left	3	n	n	2.59	1.41							2.4	1.2	10.21	22.1	n
3_508	5	10.4	1.84	4.56	1.68	2.56	2.72	3.68	2.37			3.39	2.35	17.59	37.61	46.46
3_543 left	5	n	n	3.68	2.29	3.76	2.48	3.09	2.11			2.93	2.4	11.46	22.82	34.6
3_543 right	4	5.44	1.63	3.04	2.05	2.88	1.47					2.21	1.84	6.16	14.71	23.75
3_565	5	n	n	4.37	1.95	3.23	1.87	4.24	2.29			2.72	2.19	11.99	27.24	32.89
3_581	5	1.44	1.73	2	1.57	2.35	1.65	1.97	1.79			1.71	1.71	8.13	18.39	22.36
3_590	4	7.17	2.19	3.49	1.76	2.27	1.87					2.59	1.84	11.99	25.11	28.89
3_646	4	7.04	1.76	5.78	1.39	3.81	1.71					2.67	1.6	10.69	22.2	33.37
3_648	2	n	n	1.47	1.2							2.03	1.92	10.1	26.07	n
3_654	5	4.8	1.81	3.25	1.39	2.64	1.92	2.77	1.81			2.61	1.84	8.8	21.43	29.88
3_680	4	n	n	1.52	1.39	2.35	2.51					1.81	2.03	11.46	23.43	n
3_697	4	4.85	1.79	5.17	1.87	3.17	1.71					2.43	1.76	12.07	22.74	25.46
3_708	3	8.64	1.65	3.6	1.81							2.11	1.52	9.78	18.34	20.63
3_714	6	n	n	6.8	1.92	4.66	2.05	4.4	2.4	3.63	2.4	2.67	2.4	17.25	34.41	60.99
3_719	5	n	n	4.26	3.73	4.29	2.85	3.44	2.67			3.55	3.57	18.58	36.06	63.71
3_722	3	6.29	1.52	2.43	1.73							2.67	2.19	8.74	21.59	19.83
3_723	5	n	n	2.72	1.52	1.89	1.65	2.61	2.43			2.03	1.84	11.38	23.67	38.38
3_726	3	3.68	1.52	2.45	1.28							2.35	1.09	6	12.23	15.91
	**Openings**	**HVL**	**HVW**	**IVL**	**IVW**							**TVL**	**TVW**	**ER**	**EW**	**TotalL**
**Average:**	4.44	6.77	1.95	3.33	2.09							2.43	1.88	11.73	24.46	34.27
**min**	2	1.44	0.91	1.47	0.99							1.25	0.95	5.46	12.23	
**max**	11	19.64	5.62	6.8	4.21							3.86	3.57	22.18	43.21	
**st dev**		3.53426	0.75727	0.93797	0.58576							0.51908	0.45577	3.33479	6.398816	14.5459
