# Supplementary material for: Complex sublinear burrows in the deep sea may be constructed by amphipods
Source: Ecol Evol. 2023 Mar 16;13(3):e9867. doi: 10.1002/ece3.9867 (PMC10018091; doi:10.1002/ece3.9867)
Supplement: Supplementary file 3 — Table S3 [file ECE3-13-e9867-s002.pdf]

Supplementary Table S3. List of all 146 burrows from still images and identified associated animals species.

Burrow ID	Measured	Fig.	Number of Valves	Maerid Amphipod	Amphipod indet.	Munnopsid Isopod	Demostomatid Isopod	Isopod indet.	*Munidopsis*	Shrimp	Aspidodiadematid Urchin	Irregular Urchin	*Abyssocucumis*	*Elpidia*	Annelid Tube	Glass Sponge
1_106 left	Y	3c	4	1												
1_106 right	N		7													
1_140	Y		4													
1_231	N		4													
1_252	Y		4													
1_260	Y		4	1												
1_270	Y		6			2										
1_305	Y		6					1								
1_307	Y		5													
1_309	Y		5			1										
1_311	Y		7		1											
1_361 left	N		4													
1_361 right	Y		5													
1_365	N		4		1											
1_37	N		4													
1_414	Y		5		1											
1_420	N		4													
1_432	N		3					1								
1_444	Y		8								1				1	
1_482	Y		3					1								
1_486	N		4												1	
1_507	Y	2b	2				1									
1_517	N		6		1										2	
1_554	Y		8													
1_567	N		3													
1_571	N		3													
1_580	Y		4		1											
1_599	Y		4												1	
1_609	Y		4			2										
1_617 left	N		4													
1_617 right	N		5					1								
1_618	Y		4													
1_622	Y		5					1								
1_624 bottom	N		4													
1_624 top	N		4													
1_629	Y		3													
1_633	N		3													
1_640	Y		n													
1_645	N		3													
1_664	Y		2													
1_68	Y		6					1								
1_683	N		5													
1_685	Y		4												1	
1_729 left	Y		6												1	
1_729 right	N		5													
1_73 left	N		5													
1_73 right	Y		7													
1_731	Y		5			2										
1_76	N		3													
1_762	Y		4													
1_767	N		5													
1_774	Y		3					1								
1_785	N		3													
1_79	N		5													
1_92	Y		3													
1_94	Y		4													
1_96	N		6					1								
2_114	N		4													
2_166	N		4													
2_258	Y		3													
2_265	Y	2g	4										1		2	
3_100	Y	3d	3	1							1					
3_105	N		8												1	
3_161	N		5			1									1	
3_202	Y		6													
3_211	Y	2d-e	3								1				1	
3_220	N		11									1			1	
3_226	N		3			1										
3_233	Y		4								1					
3_250	Y		5												1	
3_274	N		n					1			1					
3_281	N		2		1			2							1	
3_304	N		3													
3_307 left	N		4													
3_307 right	Y		5													
3_313	Y		5													
3_317 bottom	Y		7													
3_317 top	N		5						1							
3_321 left	N		4													
3_321 right	N		2													
3_330	Y		3		1											
3_352 bottom	Y	2c	6													1
3_352 top	N		n													
3_365	N	2f	5		1							1				
3_384	Y		3													
3_394	Y		5												1	
3_406	N		6									1				
3_415	Y		3		1											
3_421	Y		4												1	
3_443	N		4													
3_45	Y		5													
3_453 left	N		3													
3_453 right	N		3													
3_461	Y		4		1	2				2						
3_465	N		6													
3_470	Y		3													
3_482	N		6			2										
3_488 bottom	N		9			2										
3_488 top	N		2													
3_492 botom	Y		4													
3_492 top	N		n													
3_493 left	Y		3			1										
3_493 right	N		5													
3_497 left	N		6													
3_497 right	N		2													
3_508	Y		5			1										
3_51	N		5													
3_53	Y		8					1								
3_538 left	N		n			1										
3_538 right	N		3		1											
3_543 left	Y		5													
3_543 right	Y		4												1	
3_547	N		4													
3_549	N	3a-b	7	1												

Supplementary Table S3. List of all 146 burrows from still images and identified associated animals species.

Burrow ID	Measured	Fig.	Number of Valves	Maerid Amphipod	Amphipod indet.	Munnopsid Isopod	Demostomatid Isopod	Isopod indet.	*Munidopsis*	Shrim p	Aspidodiadematid Urchin	Irregular Urchin	*Abyssocucumis*	*Elpidia*	Annelid Tube	Glass Sponge
3_565	Y		5													
3_581	Y		5								1					
3_583	N		n													
3_589	N		6													
3_590	Y	3e	4	1												
3_591 left	N		4													
3_591 right	N		10		1							1		1		
3_62	N		5													
3_628	N		6													
3_632	N		n			1										
3_638 left	N		3													
3_638 right	N		6								1					
3_639	N		5													
3_646	Y		4		1											
3_648	Y		2													
3_654	Y		5													
3_676	N		4													
3_680	Y		4													
3_697	Y	2a	4		1	2										
3_70	Y		3													
3_708	Y		3													
3_714	Y		6												1	
3_717	N		6													
3_719	Y		5		1	1										
3_722	Y		3								1					
3_723	Y		5													
3_726	Y		3													
3_79	Y		5		1											
3_8	N		4													
3_83	N		4											1	2	
3_86	N		5													
3_98	Y		4					1								
**No. Inds. Seen**				**5**	**16**	**22**	**1**	**13**	**1**	**2**	**8**	**4**	**1**	**2**	**21**	**1**
**No. Instances**				**5**	**16**	**15**	**1**	**12**	**1**	**1**	**8**	**4**	**1**	**2**	**18**	**1**
