# Supplementary material for: Complex sublinear burrows in the deep sea may be constructed by amphipods
Source: Ecol Evol. 2023 Mar 16;13(3):e9867. doi: 10.1002/ece3.9867 (PMC10018091; doi:10.1002/ece3.9867)
Supplement: Supplementary file 4 — Table S4 [file ECE3-13-e9867-s004.docx]

**Supplementary Table S4**. List of amphipod and isopod species documented to make persistent burrow structures or mud based tubes in sediment. This excludes other species that dig holes in other substrata (kelp) or that make burrowing movement in sediment while searching for food.

| **Species** | **Family** | **Distribution depth of species** | **Reference for burrowing behaviour** |
| --- | --- | --- | --- |
| **Amphipoda** |  |  |  |
| *Autonoe longipes* (Liljeborg, 1852) | Aoridae | 20-375 m | Enequist, 1949 |
| *Casco bigelowi* Blake, 1929 | Cheirocratidae | subtidal | Thiel, 1999 |
| *Cheirocratus sundevallii* (Rathke, 1843) | Cheirocratidae | 0-200 m | Enequist, 1949 |
| *Corophium arenarium* Crawford, 1937 | Corophiidae | 10-60 m | Crawford, 1937 |
| *Corophium volutator* (Pallas, 1766) | Corophiidae | intertidal/mudflats | Thamdrup, 1935; Watkin, 1947 |
| *Leptocheirus pinguis* (Stimpson, 1853) | Corophiidae | 10-260 m | Thiel, 1999 |
| *Eriopisa elongate* (Bruzelius, 1859) | Eriopisidae | 30-800 m | Enequist, 1949 |
| *Maera loveni* (Bruzelius, 1859) | Maeridae | 20-1220 m | Enequist, 1949 |
| *Paraceradocus gibber* Andres, 1984 | Maeridae | 10-350 m | Coleman, 1991 |
| *Neohela monstrosa* (Boeck, 1861) | Unciolidae | 150-2200 m | Enequist, 1949; Buhl-Mortensen et al, 2016 |
| **Isopoda** |  |  |  |
| *Ischnomesus bispinosus* (G.O. Sars, 1868) | Ischnomesidae | 140-210 m | Hessler & Strömberg 1989 |
| *Macrostylis spinifera* G.O. Sars, 1899 | Macrostylidae | 140-210 m | Hessler & Strömberg 1989 |
| *Pseudarachna hirsuta* G.O. Sars, 1899 | Ilyarachnidae | 140-210 m | Hessler & Strömberg 1989 |

**References**

Buhl-Mortensen, L., A.H.S. Tandberg, P. Buhl-Mortensen and A.R Gates. 2016. Behaviour and habitat of *Neohela monstrosa* (Boeck, 1861) (Amphipoda: Corophiida) in Norwegian Sea deep water. Journal of Natural History 50:323-337. <http://dx.doi.org/10.1080/00222933.2015.1062152>

Coleman, Ch. O. 1989. Burrowing, Grooming, and Feeding Behaviour of *Paraceradocus*, an Antarctic Amphipod Genus (Crustacea). Polar Biology 10: 43-48.

Crawford, G.I. 1937. A review of the amphipod genus *Corophium* with notes on the British Species. Journal of the Marine Biological Association of the United Kingdom 21: 589-630.

Enequist P. 1949. Studies on the soft-bottom amphipods of the Skagerak. Zool Bidragen Uppsala 28:299-492.

Hessler, R., Strömberg, J.-O. 1989. Behavior of janiroidean isopods (Asellota), with special reference to deep-sea genera. Sarsia. 74. 145-159.

Thamdrup, H.M. 1935. Beiträge zur Ökologie der Wattenfauna auf experimenteller Grundlage. Meddelelser fra Kommissionen for Danmarks Fiskeri- og Havundersøgelser (serie: Fiskeri) 10: 1-125.

Thiel, M. 1999. Extended parental care in marine amphipods II. Maternal protection of juveniles from predation. Journal of Experimental Marine Biology and Ecology 234: 235-253.

Watkin, E.E. 1947. The yearly cycle of the amphipod *Corophium volutator*. Journal of Animal Ecology 10: 77-93.
